# Using Peer Discussion Facilitated by Clicker Questions in an Informal Education Setting: Enhancing Farmer Learning of Science

**DOI:** 10.1371/journal.pone.0047564

**Published:** 2012-10-15

**Authors:** Michelle K. Smith, Seanna L. Annis, Jennifer J. Kaplan, Frank Drummond

**Affiliations:** 1 School of Biology and Ecology, University of Maine, Orono, Maine, United States of America; 2 Maine Research in STEM Education Center, University of Maine, Orono, Maine, United States of America; 3 Department of Statistics, University of Georgia, Athens, Georgia, United States of America; 4 Cooperative Extension, University of Maine, Orono, Maine, United States of America; University of Westminster, United Kingdom

## Abstract

Blueberry growers in Maine attend annual Cooperative Extension presentations given by university faculty members. These presentations cover topics, such as, how to prevent plant disease and monitor for insect pests. In 2012, in order to make the sessions more interactive and promote learning, clicker questions and peer discussion were incorporated into the presentations. Similar to what has been shown at the undergraduate level, after peer discussion, more blueberry growers gave correct answers to multiple-choice questions than when answering independently. Furthermore, because blueberry growers are characterized by diverse levels of education, experience in the field etc., we were able to determine whether demographic factors were associated with changes in performance after peer discussion. Taken together, our results suggest that clicker questions and peer discussion work equally well with adults from a variety of demographic backgrounds without disadvantaging a subset of the population and provide an important learning opportunity to the least formally educated members. Our results also indicate that clicker questions with peer discussion were viewed as a positive addition to university-related informal science education sessions.

## Introduction

Having students respond to multiple-choice format questions designed to test conceptual understanding using personal response systems or “clickers” is one strategy that has been used to promote interaction and learning in K-12 and undergraduate courses (reviewed in [1], [2]). Instructors who use clickers and clicker questions often pair that use with an approach called peer instruction. This approach encourages students to verbalize their thinking and interact with their peers to arrive at an answer [3]. In one commonly used mode of peer instruction, students first answer a concept question individually, then discuss the question with peers, and finally resubmit the response, all before the answer to the question is revealed. The instructor then discusses the answer choices and often shows a bar chart of the student responses. The bar chart of student results gives both instructors and students immediate feedback on how well a concept is understood.

Work at the undergraduate level has shown that students are more likely to answer a question correctly after peer discussion [3]–[5]. Furthermore, studies that use pairs of matched questions determined that students learn from discussing clicker questions with their peers [4], [6] and this interactive technique is especially effective when peer discussion is followed by instructor explanation [5].

Conceptual questions and clickers encourage active learning in formal education settings, and can also be used in informal education settings, for example, in courses targeted towards non-student adults. Although there have been anecdotal reports that concept questions and clickers work well in these settings, to our knowledge, no one has reported on whether adults not in a formal academic setting benefit from answering and discussing questions with their peers and whether specific demographic variables, such as age, sex, and education level are associated with changes in performance after peer discussion.

In this study, we investigated whether there is evidence that peer discussion is valuable for adult learners in informal settings using a population of farmers who grow wild blueberries in Maine [7]. These blueberry growers attend an annual “Blueberry School” that is structured as a series of cooperative extension lecture-style presentations given by faculty members of the University of Maine Agricultural and Forestry Experiment Station and the University of Maine Cooperative Extension. In order to make the Blueberry School presentations more interactive, clicker questions and peer discussion were incorporated into the talks in March, 2012. This modification is aligned with a growing movement to redesign cooperative extension presentations so that traditional lecture methods, where university faculty present information, are de-emphasized and group learning is promoted [8], [9]. One reason for this proposed shift is that if social interaction is fostered, farmers will be more likely to contribute local knowledge to the group because they perceive the cooperative extension sessions as a more welcoming environment [10].

**Figure 1:**
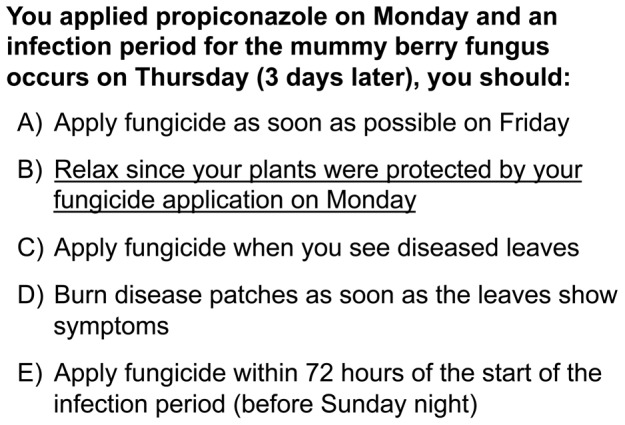
Example clicker question about the timing of fungicide applications to control mummy berry disease used in the cooperative extension sessions. The correct answer is underlined.

**Figure 2:**
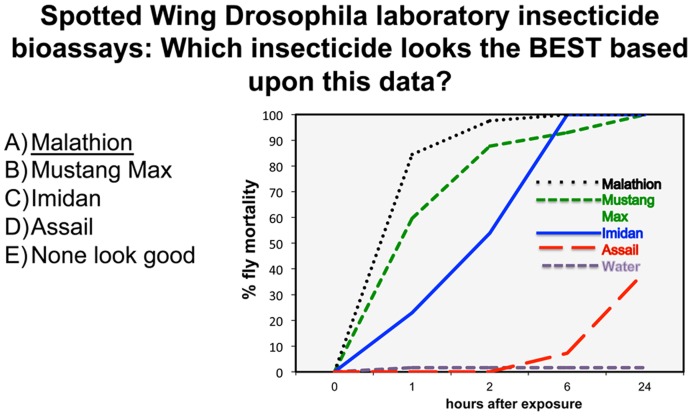
Clicker question about the best insecticide to use to control Spotted Wing *Drosophila*. This question, which focuses on graph interpretation, was the lowest scoring question (Q-I = 49%, Q-AD = 63%). The correct answer is underlined.

The clicker questions that were added to the Blueberry School presentations focused on practical scenarios, such as, how frequently to apply fungicide for avoiding a common disease while still encouraging cost savings (Figure 1) and how to interpret a graph comparing insecticide effectiveness against a newly invasive *Drosophila* species (Figure 2; the text of all content questions is shown in Figure S1). When possible, incorrect answer choices were written based on incorrect ideas stated by blueberry growers during previous interactions. Participants were also asked several demographic questions (the text of all demographic questions is shown in Figure S2), so the effectiveness of peer discussion could be evaluated in different demographic groups.

This study investigates several questions regarding the use of clickers in informal education settings for adults including: 1) Does peer discussion improve performance of adult learners answering questions with clickers in an informal science setting? 2) Do members of a mixed demographic group benefit from peer discussion? and 3) Are clicker questions viewed as a positive addition to university-related informal science education?

## Materials and Methods

### Research Environment

The University of Maine Blueberry Schools are offered at three locations in Maine (Waldoboro, Ellsworth, and Machias) in mid-March every year. The Blueberry Schools meet once a year for three hours and attendees are awarded Maine Pesticide Applicator credits for attending the presentations at one of the locations.

There were a total of five to seven speakers at each class location in 2012. Authors S.A. and F.D. each spoke for 30 minutes and were the only speakers to use a combination of clicker questions and peer discussion during their presentations. One additional speaker used the clickers to individually poll blueberry growers’ opinions on management strategies before and after his presentation. The data from the additional speaker are not included in this study.

S.A. and F.D. are tenured faculty who do research on blueberry plant diseases and insect pests, respectively. S.A has given presentations at the Blueberry School for nine years and F.D. has given presentations for 22 years. Before giving the presentations reported on in this paper, S.A. was familiar with using clickers and had taught with them recently in her University of Maine non-majors biology course. F.D. had never taught with clickers before.

### Participation Rates and Demographic Information

All blueberry growers were given a clicker, but were told that participation was optional both during questions that focused on content information in the presentations and for the demographic questions. In addition, the blueberry growers were told that their responses were anonymous and that an individual could not be traced to a specific clicker serial number. We decided to keep the blueberry growers responses anonymous as this is the standard procedure used by the University of Maine Cooperative Extension for all surveys of farmers. Based on our counts of attendance versus the number of people voting on clicker questions, fewer than 5% of the blueberry growers opted out of voting with clickers during the presentation. The number of blueberry growers participating with clickers in each session were as follows: 24 in Waldoboro, 34 in Ellsworth, and 48 in Machias.

Demographic information on the blueberry growers who participated is shown in Table 1. We use the term ‘grower’ in our study to represent an audience that was primarily comprised of blueberry farmers managing their own land, but also included spouses of farmers, hired farm-workers, managers of blueberry land owned by others, blueberry land owners that do not actively farm the land, and individuals that work for companies that provide services to blueberry farmers and so have a variety of roles in the blueberry industry.

**Table 1:** Blueberry grower demographic information.

Category	Subcategory	Result (%)
Sex	Male	84
	Female	16
Age	Under 40	9
	40 and older	91
Level of education	High school	26
	Some college	27
	College degree	33
	Graduate degree	14
Household income derived from blueberries	>90%	30
	25–75%	36
	<25%	34
Role on farm	Grower, own land	42
	Manager, not land owner	12
	Grower and manager	27
	Landowner only	7
	Other	12
Time worked with blueberries	<10 years	20
	11–30 years	35
	>30 years	45

### Procedure for Recording Clicker Question Answers

Each of the presentations by S.A. and F.D. included three content questions for a total of six questions (Figure S1). The clicker questions focused on practical scenarios the growers would encounter on the farm and the incorrect answers were based on information or thinking the presenters have heard over years of working with blueberry growers. In preparation for the Blueberry School presentations, S.A. and F.D. gave several practice talks to audiences comprised of University of Maine faculty, graduate students, and research assistants who regularly work with blueberry growers. Based on feedback, the clicker questions were modified both for scientific accuracy and clarity.

During the Blueberry School presentation, the peer instruction method [3] was used for each question. For each of the content questions, blueberry growers would see the question projected on a screen, the presenter would read the question aloud to minimize reading level issues, and then each blueberry grower would respond on his/her own. After the individual votes were recorded, the bar chart of answers was kept hidden and the blueberry growers were encouraged to talk about the question with their neighbors and vote again. The presenters set the timer on the clicker software so it revealed the elapsed time after the question was posed to the audience. On average, the individual vote was open for 73 seconds (STD = 16.5 seconds) and the time for the group discussion and post discussion vote was 101 seconds (STD = 10.1 seconds). The voting times per question along with the number of blueberry growers voting for each question are shown in Figure S3. For the individual vote, when ∼75% of the votes were recorded, the presenters made an announcement that the blueberry growers should select their final answer choices. For the group discussion, the presenters asked audience members to talk and then ∼45 seconds later began requesting that the blueberry growers select an answer choice after they had discussed the questions with their peers. Once ∼75% of the votes were recorded, the presenters asked for any last votes.

Results from questions answered before peer discussion are labeled Q-I (for Question-Individual Vote) and questions answered after peer discussion are labeled Q-AD (for Question-After Discussion vote). The data set included responses from 106 blueberry growers (∼20% of the blueberry growers in the state of Maine). For data from any one question from an individual blueberry grower to be included in our study, the blueberry grower had to answer both Q-I and Q-AD. If, for example, a blueberry grower answered Q-AD but not Q-I for a question, the data point was removed for that blueberry grower for that question. Most growers answered both Q-I and Q-AD for five or six of the six questions (Table 2).

**Table 2:** Frequency of questions where both Q-I and Q-AD were answered by the blueberry growers.

Number of Questions where both Q-I and Q-AD Answered	Percent Blueberry Growers
1	4
2	11
3	15
4	12
5	25
6	33

The demographic questions (Figure S2) were only answered by individual votes, and results of the demographic information were not revealed to the participants.

The content and demographic clicker questions were the same at all three Blueberry School locations. Two of the clicker questions were focused on information blueberry growers had seen in previous years and at the first class, the growers were asked to answer these questions immediately after the relevant information was given. As a result, over 90% of the blueberry growers answered the question correctly before talking to their peers. In the second and third class, we moved each of these questions to precede the presentation of the specific information pertinent to answer these questions. In addition, all data from the three Blueberry Schools were pooled due to sample size considerations. Analyses of the data, therefore, were not aimed at ascertaining the effect of growing region or its interactions with demographic factors on grower performance.

### Statistical Analyses

The change in learning between answers within question sets was computed for each blueberry grower using a modified version of the Hake normalized gain formula [11] known as normalized change <c> [12]. Normalized change values provide a measure of how much a blueberry grower’s performance changes compared with that individual’s maximum possible change. When calculating the mean normalized change between Q-I and Q-AD over all question sets for a given blueberry grower, the following formula was used when an individual’s mean Q-AD score was higher than the mean Q-I score (most cases): <c> = 100[(mean Q-AD − mean Q-I)/(100 − mean Q-I)]. Alternatively, if an individual’s mean Q-I score was higher than the mean Q-AD score, <c> = 100[(mean Q-AD – mean Q-I)/(mean Q-I)], was used. In cases where an individual’s mean Q-I score and the mean Q-AD score equaled either 100 or 0, the normalized change score for that blueberry grower was not calculated, because otherwise <c> would be recorded as 0. In the following analyses, the blueberry grower is the unit of repetition and the mean Q-I score, mean Q-AD score or normalized gain for all questions answered by an individual grower are the measurements analyzed.

A logistic regression model was used to examine whether specific demographic variables impacted peer discussion among blueberry growers. Logistic regression models provide an extension of multiple regression models when the response variable is a binary variable (having two distinct categories). For this model, each grower was initially classified as: increasing his/her overall score after discussion (increase), showing no overall change before and after peer discussion (no change), decreasing his/her score after discussion (decrease) or having a perfect overall score before and after peer discussion (ceiling). For the logistic regression model analysis, the four classification categories (increase, no change, decrease, and ceiling) were collapsed into two categories: 1) those who were advantaged by talking to their peers (increased) and 2) those who were not (decreased and no change combined). Data from blueberry growers in the ceiling category were not used in this analysis. The two categories used as the response variable in the logistic model were advantage, coded as 1 and no advantage, coded as 0. The demographic variables listed in Table 1 were used as factors. Time worked with blueberries, age, household income derived from blueberries, and education were coded as ordinal variables, meaning that while the data were collected as categorical variables, there is an order inherent to the categories; gender and role on farm were coded as nominal variables.

All data summaries and statistical analyses were performed with JMP (Cary, NC) or Excel (Microsoft, Redmond, WA).

### Institutional Review Board Statement

Approval to evaluate blueberry growers’ responses to clicker questions (exempt status, protocol no. 2012-04-05) was granted by the Institutional Review Board at the University of Maine.

## Results

Overall the blueberry growers answered more questions correctly after peer-discussion (Q-AD) than before (Q-I) (Table 3). This difference is significantly different when comparing either the paired difference of total percent correct for each blueberry grower (Q-AD – Q-I, paired t-test, t_105_ = 7.11, p<0.0001) or the normalized change scores (<c>, t-test, t_85_ = 6.95, p<0.0001). Aside from the individuals who had either none or all of the questions correct both before and after discussion, the distribution of the individual blueberry growers’ scores ranged from 20% to 80% correct. This range was the same before and after discussion, but the scores shifted upwards after peer discussion, presenting a left skewed distribution after discussion as compared to a more symmetric distribution before.

**Table 3:** Summary statistics for performance variables for all blueberry growers.

Variable	n	Mean(%)	STD(%)	SEM(%)
Q-I	106	55.3	31.74	3.083
Q-AD	106	71.8	27.21	2.643
Raw Difference	106	16.4	23.79	2.379
Normalized ChangeScore <c>	91	35.4	41.46	4.346

**Figure 3:**
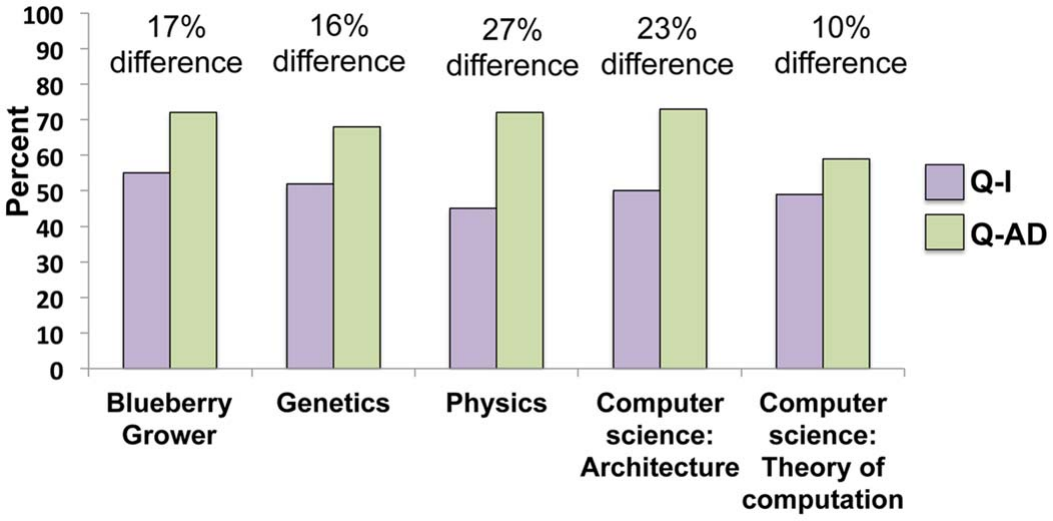
A comparison of blueberry grower and undergraduate science student performance on clicker questions: before (Q-I) and after peer discussion (Q-AD). Data from the other science classes is taken from published studies, see text for details.

The Q-I and Q-AD results from the blueberry growers were compared with other published reports on undergraduate student clicker question performance in a variety of science courses including genetics, physics, and computer science courses (Figure 3) [4], [6], [13]. The performance patterns are similar for all groups with higher levels of correct answers after peer discussion and the increase between Q-I and Q-AD ranging from 10–27%.

**Figure 4:**
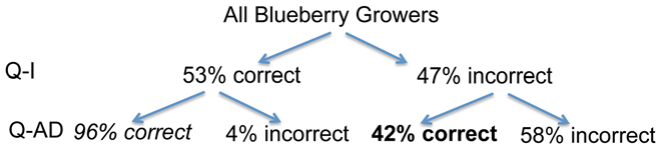
Breakdown of blueberry grower answer patterns.

When comparing votes before and after discussion, four outcomes are possible, a blueberry grower can: 1) answer Q-I and Q-AD correctly, 2) answer Q-I correctly and Q-AD incorrectly, 3) answer Q-I incorrectly and Q-AD correctly, 4) answer Q-I and Q-AD incorrectly. The patterns of behavior for blueberry growers over all six questions shows 96% of blueberry growers who answered Q-I correctly answered Q-AD correctly after peer discussion (Figure 4, number in italics). In contrast, 42% of the blueberry growers who answered Q-I incorrectly go on to correctly answer Q-AD after peer discussion (Figure 4, number in bold).

**Figure 5:**
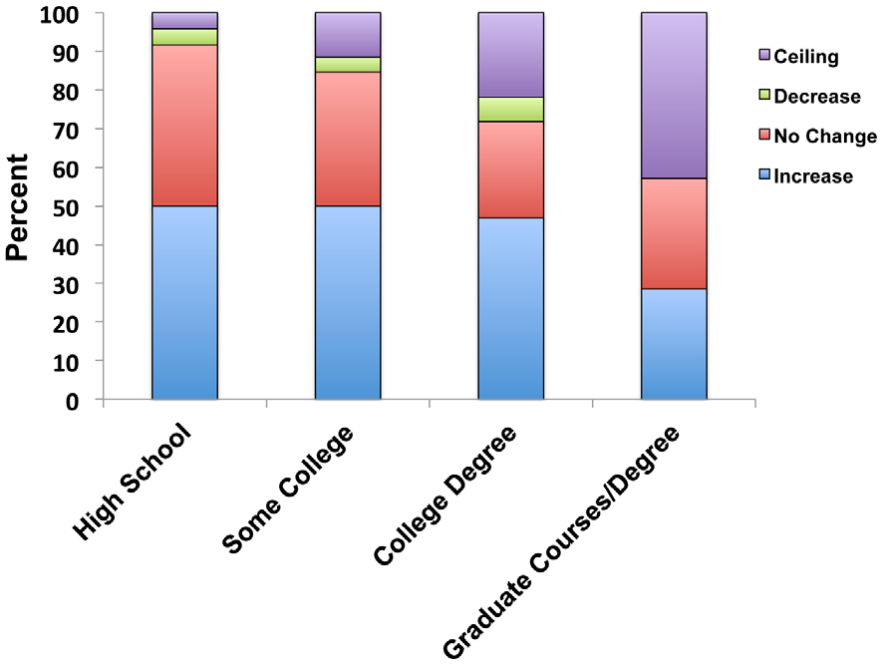
Performance on clicker questions by blueberry growers grouped by their education level. Performance within each education level was divided according to four classifications: increasing his/her overall score after discussion (increase), showing no overall change before and after peer discussion (no change), decreasing his/her score after discussion (decrease), or having a perfect score before and after peer discussion (ceiling).

Demographic question results revealed that blueberry growers have a diverse level of education (Table 1), and about a quarter of the growers have no formal education beyond high school. To determine if education level is associated with performance on clicker questions, we analyzed the performance results by this demographic variable. Specifically, each grower was classified as: increasing his/her overall score after discussion (increase), showing no overall change before and after peer discussion (no change), decreasing his/her score after discussion (decrease) or having a perfect overall score before and after peer discussion (ceiling). Groups of growers with a high school education, some college, or a college degree all have similar percentages of people who increase their scores after peer discussion (Figure 5). Furthermore, a statistical comparison among all education level groups revealed no significant difference in the distribution of growers in the increase, no change, decrease, and ceiling categories across all education levels (Fisher’s 4×4 exact test, p = 0.237). These results suggest that blueberry growers with high school degrees had comparable experiences to growers from the other education groups. The high school education group, however, is the least likely to have participants in the ceiling category, indicating that they have the most opportunity to learn from discussing questions with peers.

The data also were sorted by other demographic variables including sex, age, percentage of income coming from blueberries, role on the farm (grower, manager, landowner), and number of years working with blueberries. Fisher’s exact tests revealed no significant differences in the distribution of blueberry growers in the increase, no change, decrease, and ceiling groups when they were sorted by any of the demographic variables (all results p>0.10, Table S1). Taken together, these results suggest that on the whole blueberry growers have a comparable experience with regard to the use of clickers and peer instruction regardless of their demographic classification.

The above analyses consider the effect of each demographic variable on the outcome of peer instruction independently; therefore, we also performed a logistic regression analysis to incorporate all of the demographic variables into one model. Prior to the use of logistic regression, Chi-squared tests or Fisher’s exact tests, when the data were insufficient to meet the sample size qualification for a Chi-squared test, were used to investigate pairwise associations between the demographic variables listed in Table 1. The role of the blueberry grower had a statistically significant association with all other demographic variables, and so this variable was dropped from the model. Additional statistically significant associations between demographic variables that remained in the model are listed in Table 4. These statistically significant associations, while important to note, were generally expected. For example, the time that a blueberry grower had been working with blueberries was associated with age. Therefore, it is reasonable to assume that the relationships among demographic variables in the sample reflect relationships among the population of Maine blueberry growers, and do not affect the applicability of the regression analysis. These relationships would, however, require further interpretation if any of them were found to be significant predictor variables in the logistic regression model.

**Table 4:** Significant associations between demographic variables in the ordinal logistic analysis.

Correlation	?^2^	df	p value	Result
Time worked with blueberries and age	12.2	2	0.0022	Older blueberry growers were more likely to have been in the blueberry business longer than the younger blueberry growers
Time worked with blueberries and household income derived from blueberries	11.6	4	0.0207	Blueberry growers newer to the business were more likely to derive a higher percent of their income from blueberry farming when compared to blueberry growers who have been in the business longer
Time worked with blueberries and sex	9.48	2	0.0087	Women were more likely than men to be newer to blueberry farming
Household income derived from blueberries and level of education	18.2	6	0.0059	Blueberry growers with higher levels of education tend to derive a higher percentage of their income from blueberry farming

The response variable for the model comprised the two categories: 1) blueberry growers who were advantaged by talking to their peers (increased Q-I to Q-AD) and 2) blueberry growers who were not (decreased Q-I to Q-AD and no change combined). The logistic regression analysis simultaneously controls for the relationship of each demographic variable to both the benefit or lack of benefit of peer discussion. The results of the analysis showed that none of the demographic variables had a significant relationship with a blueberry grower’s status of being advantaged or not by the peer discussion activities (model χ^2^_9_ = 3.68, p = 0.9311; p-values for the individual factors: 0.3923<p<0.8577, based on 0.121< χ^2^ values <0.765, full results in Table S2). In addition, neither selecting nor deleting one variable at a time from the model produced a significant relationship between any demographic variable and the advantage of peer discussion to the blueberry grower, further supporting the result that all blueberry growers, regardless of demographic factors, share similar advantages of peer discussion. Because none of the demographic variables were shown to be significant predictors of a blueberry grower having an advantage during peer discussion, no further analysis of the associations between the demographic variables was necessary.

## Discussion

### Do Adults in an Informal Science Education Setting Benefit from Discussing Clicker Questions with their Peers?

When clickers were introduced into informal science education sessions for blueberry growers, they participated, engaged in peer discussion, and improved their scores after talking with peers. Notably, growers show performance gains similar to what has been reported for students in undergraduate courses (Figure 3) [4], [6], [13]. This result suggests that older adults in informal science settings respond to educational paradigms in a manner similar to that exhibited by undergraduates in more formal settings. Furthermore, our observations during the presentations revealed that the blueberry growers were motivated to answer questions correctly and were very competitive about being correct, with some shouting “yes!” or giving out high-fives when the presenter revealed the correct answer.

Instructors using clicker questions at the K-12 and undergraduate levels use a variety of strategies for encouraging students to answer clicker questions [2]. These strategies range from giving participation points for any answer, justifying this with the idea that clicker questions will help students practice for exams, to grading student responses for correctness. Given that the blueberry growers were not receiving academic credit and their responses were anonymous, why would they try to get the correct answer? One explanation is that the blueberry growers’ profits depend on understanding the information in the presentations, so just as students are motivated to answer clicker questions to prepare for the exam, blueberry growers are motivated to answer questions correctly to have a productive growing season. Furthermore, a recent survey of farmers who participate in cooperative extension courses indicated that farmers are most strongly motivated to learn in these courses by desires to save time and money, learn about cutting-edge research, and access the social aspects of agriculture [14]. The clicker questions were aligned with all three of these motivations, asking about both practical situations focused on cost-saving and current research, and encouraging growers to respond in a social way. Another explanation is that the blueberry growers were motivated because they viewed clicker questions as a fun game. As one grower commented: “it [using clickers] makes it fun to test what we think we know against what we really know.” Future work will focus on why adults in informal education settings are motivated to answer clicker questions correctly. Furthermore, we will explore this question in a variety of adult courses, such as university extension courses that focus on other types of agriculture and courses that focus on hobbies such as gardening, to determine whether those motivations change depending on whether the information is linked to income.

### Do Presenters in Informal Science Education Settings Need to be Concerned About Asking Clicker Questions to an Audience with Variable Demographic backgrounds?

To our knowledge, few studies have examined whether demographic variables impact performance on clicker questions. One study examined differences in answering clicker questions among male and female chemical engineering undergraduate students and found that females participated in clicker questions more regularly than males, but for males there was a stronger relationship between active participation and grade improvement [15]. Another study that focused on undergraduate introductory physics students found that in classes where clickers were used, there were no significant differences between pre- and post-test gains on the Conceptual Survey of Electricity and Magnetism (CSEM) for males and females, but in classes where clickers were not used, males showed significantly larger learning gains on the same assessment [16]. The authors suggested that the women may feel more comfortable participating anonymously with clickers and would consequently learn more in the course.

Because the background literature on the effects of clicker use for various demographic variables is sparse and blueberry growers are comprised of people with large variations in several demographic variables (Table 1), it was important that our results were analyzed by demographic groups to ensure that adding clicker questions and peer discussion to adult informal science presentations was not disenfranchising any particular group. Notably, there was no significant effects of any demographic classification (education level, age, sex etc.) on the percentage of growers in groups who increased their scores after discussion (increase), showed no change before and after peer discussion (no change), decreased their scores after discussion (decrease), or had perfect scores before and after peer discussion (ceiling) (Figure 5 and data not shown). This result also held true when all of the demographic variables were analyzed together in a logistic regression model. In addition, our results show that members of the group with the lowest education level were the least likely to be at the ceiling level and, therefore, had the greatest opportunity to learn from their peers. Taken together, the results suggest an important finding that clicker questions and peer discussion can be used with adults with diverse demographic backgrounds without disadvantaging a subset of the population and provide an important learning opportunity to members educated at the high school level.

### What Factors are Important when Writing Clicker Questions for Adults in Informal Science Education Environments?

The questions written for these presentations (Figures 1 and 2, and the full set of questions in Figure S1) focused on practical questions the growers would encounter on the farm and the wrong answers were based on incorrect information or thinking the presenters have heard over years of working with blueberry growers. By collecting data from the growers, we were able to determine the prevalence of specific misunderstandings among the group. We also learned that blueberry growers struggle with interpreting graphs. The question shown in Figure 2, which asks blueberry growers about which pesticide does the best job of killing a newly invasive *Drosophila* species, was the lowest scoring question even after peer discussion. This result is important because many of the Blueberry School presentations given by university faculty members include information displayed in graphical form. In future years, we will be able to use clicker response data to help faculty presenters rethink how they are presenting critical information.

One concern regarding our questions is that blueberry growers who answer Q-I incorrectly are slightly less likely to change to the correct answer on Q-AD than to have the incorrect answer on Q-AD (Figure 4, number in bold). Although this pattern is similar to what has been reported in undergraduate courses [4], we noted that some questions the blueberry growers answered were more likely to move participants who were initially wrong to the correct answer. Future work will focus on determining features of clicker questions that elicit productive discussions in different types of informal science settings.

Additionally, we are interested in the long-term impact of having adults in informal science education settings answer clicker questions with peer discussion. Therefore, in future presentations we will give blueberry growers follow up questions to measure how much information they retain from previous sessions using clicker questions with peer discussion. This information will help us determine how repetitive presentations need to be from one year to the next and allow us to contribute to a growing set of literature examining whether there are long term benefits to clicker use [17]–[20].

### Are Clickers Necessary for Promoting Learning and Interaction?

Because all the blueberry growers in our study answered the questions using clickers, at this point we are unable to determine whether simply presenting the questions and discussing the information is as effective as having the blueberry growers answer questions with clickers. However, we anticipate that using clickers offers several benefits to blueberry growers based on the results of studies at the undergraduate level. Namely, these studies compare courses that do and do not use clickers and have shown that when clickers are used: 1) students learn more, 2) students are more likely to participate, and 3) instructors are better able to accurately assess student understanding [17], [21]–[23]. Future work asking adults to answer questions with and without clickers will be used to parse out the relative impact of the questions versus the use of clickers.

In addition, we will also explore whether lower-cost polling methods such as using colored cards [21], [24]–[26] can achieve similar learning results in adult informal education settings. Testing out these lower-cost methods will also allow us to explore the importance of having a device like a clicker that allows answers from adult participants to be anonymous to peers.

### What Advice did Blueberry Growers Give us?

At the end of the presentation, we asked blueberry growers: “Were the clicker questions helpful in becoming familiar with the information presented?” 89% of the blueberry growers said yes. Feedback given after the presentations included comments such as: “Some answers to the questions really had you doubting yourself which made you put a lot of thought into which answer was correct” and “It was interactive and it kept us awake.” One blueberry grower commented after the meeting that it was one of the best meetings the person had attended in 25 years. On the other hand, one blueberry grower cautioned: “Using the clicker kept me more engaged in the presentations – but be careful not to get carried away with too many clicker questions either. They do tend to slow things down, having to wait for everyone to do their clicking.” These comments suggest that, similar to formal education settings, it is important to balance disseminating information and promoting interaction.

### Conclusions

This work shows that peer discussion improves the clicker question performance of adult learners in an informal setting, all members of a mixed demographic group benefit from peer discussion of clicker questions, and clicker questions are viewed as a positive addition to a university-related informal science education sessions. In addition, our work is aligned with recent calls to transform the way university-sponsored cooperative extension courses are designed so that lecture is de-emphasized and group learning is fostered [8], [9]. Given that many adults have a distrust of science [27], it is imperative that university faculty not only transform formal university education but also work to improve informal science education. Furthermore, interactive techniques that have been shown to improve learning in K-12 and undergraduate courses can also be used to improve learning for adults.

## Supporting Information

Figure S1 Text of all content questions in the presentation for blueberry growers.
(PDF)

Figure S2 Text of all the demographic questions in the presentation for blueberry growers.
(PDF)

Figure S3 Voting times for individual and after discussion votes for each question. The error bars show STD. The number of blueberry growers participating in each question is also shown. Author S.A. asked the first three questions and author F.D. asked the last three questions.
(PDF)

Table S1 Results of Fisher’s Exact Tests of association between demographic variables and overall trend of blueberry growers in the increase, no change, decrease, and ceiling groups.
(DOCX)

Table S2 Results of the Effect Likelihood Ratio Tests by demographic variable for the Logistic Regression Model.
(DOCX)
