# Supplementary material for: Using Peer Discussion Facilitated by Clicker Questions in an Informal Education Setting: Enhancing Farmer Learning of Science
Source: PLoS One. 2012 Oct 15;7(10):e47564. doi: 10.1371/journal.pone.0047564 (PMC3471889; doi:10.1371/journal.pone.0047564)
Supplement: Figure S1 — Text of all content questions in the presentation for blueberry growers. (PDF) [file pone.0047564.s001.pdf]

Which of the following is TRUE about mummy berry disease?

A) The fungus needs a frost period to infect the plant.

B) Only leaves become infected by the fungus.

C) Leaves cannot be infected once they are open.

D) When cups show up in a field can vary each year.

E) The fungus overwinter in dead infected leaves.

You applied propiconazole on Monday and an infection period for the mummy berry fungus occurs on Thursday (3 days later), you should:

A) Apply fungicide as soon as possible on Friday

B) Relax since your plants were protected by your fungicide application on Monday

C) Apply fungicide when you see diseased leaves

D) Burn disease patches as soon as the leaves show symptoms

E) Apply fungicide within 72 hours of the start of the infection period (before Sunday night)

Question #2

Which of the following is TRUE about Valdensinia leaf spot?

A) The spores are spread to new fields by rain or wind

B) Typical burn pruning will eradicate the fungus from a field

C) After a dry spell, the infected leaves can no longer produce spores

D) The fungus survives over winter in infected leaves.

Spotted Wing Drosophila laboratory insecticide bioassays: Which insecticide looks the BEST based upon this data?

- Petri dish assays with 5 male:5 female SWD exposed to dried residues
- Mortality assessed after 1, 2, 6, and 24 hours of exposure
- Trials run on broad spectrum, reduced risk, and organic insecticides

A) Malathion
B) Mustang Max
C) Imidan
D) Assail
E) None look good

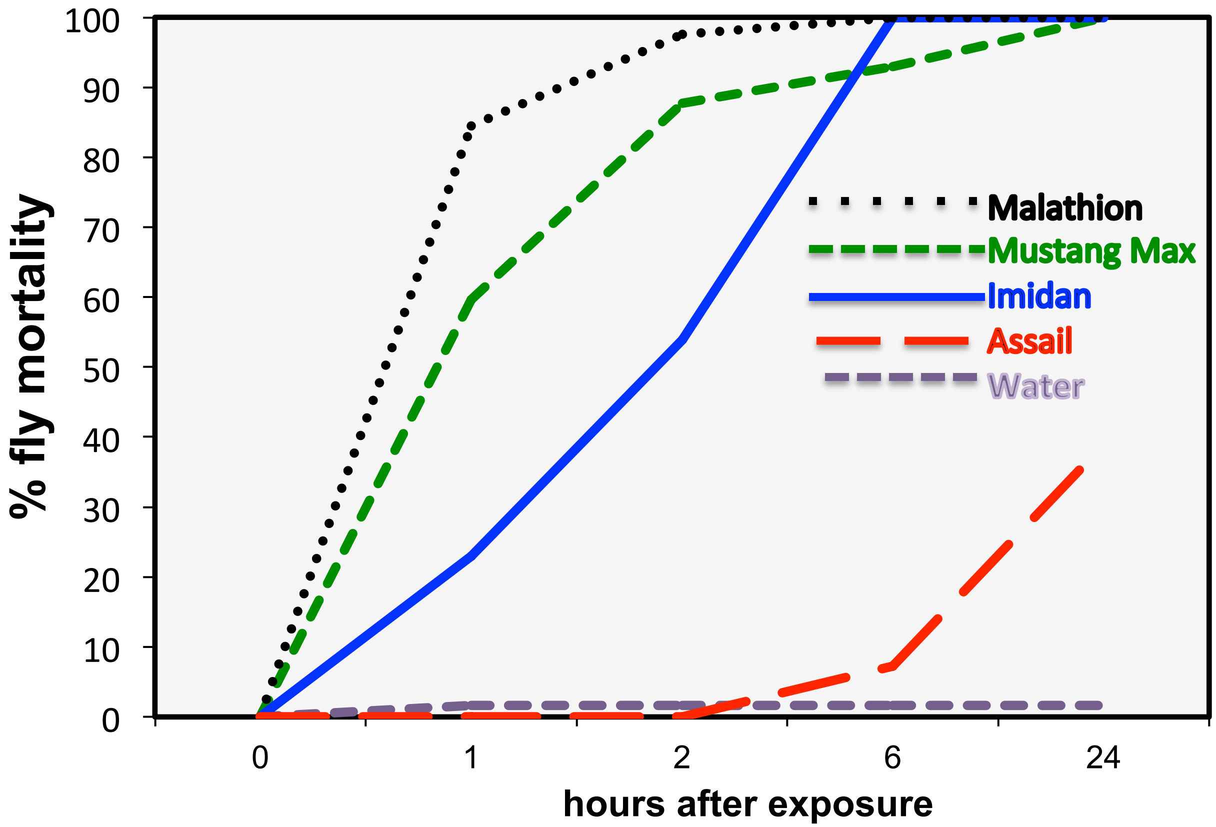

Question #4

With what you have heard tonight HOW might you reduce the amount of Spotted Wing Drosophila damage in a blueberry field?

A. use traps and spray Assail upon first fly capture
B. burn the field after harvest
C. harvest early, before traps catch adults
D. just focus on controlling the blueberry maggot fly
E. cut down all alternate fruit hosts at field edge

Question #5

Based upon what we know, what APPEARS similar between Spotted Wing Drosophila & Blueberry Maggot Fly?

A. the overwintering stage
B. the insecticides that are effective for control
C. the type of trap used for monitoring
D. the insect stage that causes the damage
E. range of fruit types that they can attack
