# Supplementary material for: Using Peer Discussion Facilitated by Clicker Questions in an Informal Education Setting: Enhancing Farmer Learning of Science
Source: PLoS One. 2012 Oct 15;7(10):e47564. doi: 10.1371/journal.pone.0047564 (PMC3471889; doi:10.1371/journal.pone.0047564)
Supplement: Figure S2 — Text of all the demographic questions in the presentation for blueberry growers. (PDF) [file pone.0047564.s002.pdf]

What is your sex and age?

A. female, younger than 40
B. female, 40 or older
C. male, younger than 40
D. male, 40 or older

What is the level of YOUR education?

A. middle school
B. high school
C. some college, no degree
D. college degree (university or community)
E. graduate courses or degree (MS or PhD)

Which best describes you?

A. I am a grower on land I own

B. I am a manager on other people's land

C. I am a grower on land I own AND a manager on other people's land

D. I am a landowner BUT NOT a grower or manager

E. Other

How long have you worked with blueberries?

A. Less than 2 years
B. 2-5 years
C. 6-10 years
D. 11-30 years
E. More than 31 years

How much of my or our (if family) income is derived from blueberries?

A. greater than 90% of income

B. about 75% of income

C. about 50% of income

D. about 25% of income

E. less than 25% of income
