# Supplementary figures and images for: Using Peer Discussion Facilitated by Clicker Questions in an Informal Education Setting: Enhancing Farmer Learning of Science
Source: PLoS One. 2012 Oct 15;7(10):e47564. doi: 10.1371/journal.pone.0047564 (PMC3471889; doi:10.1371/journal.pone.0047564)

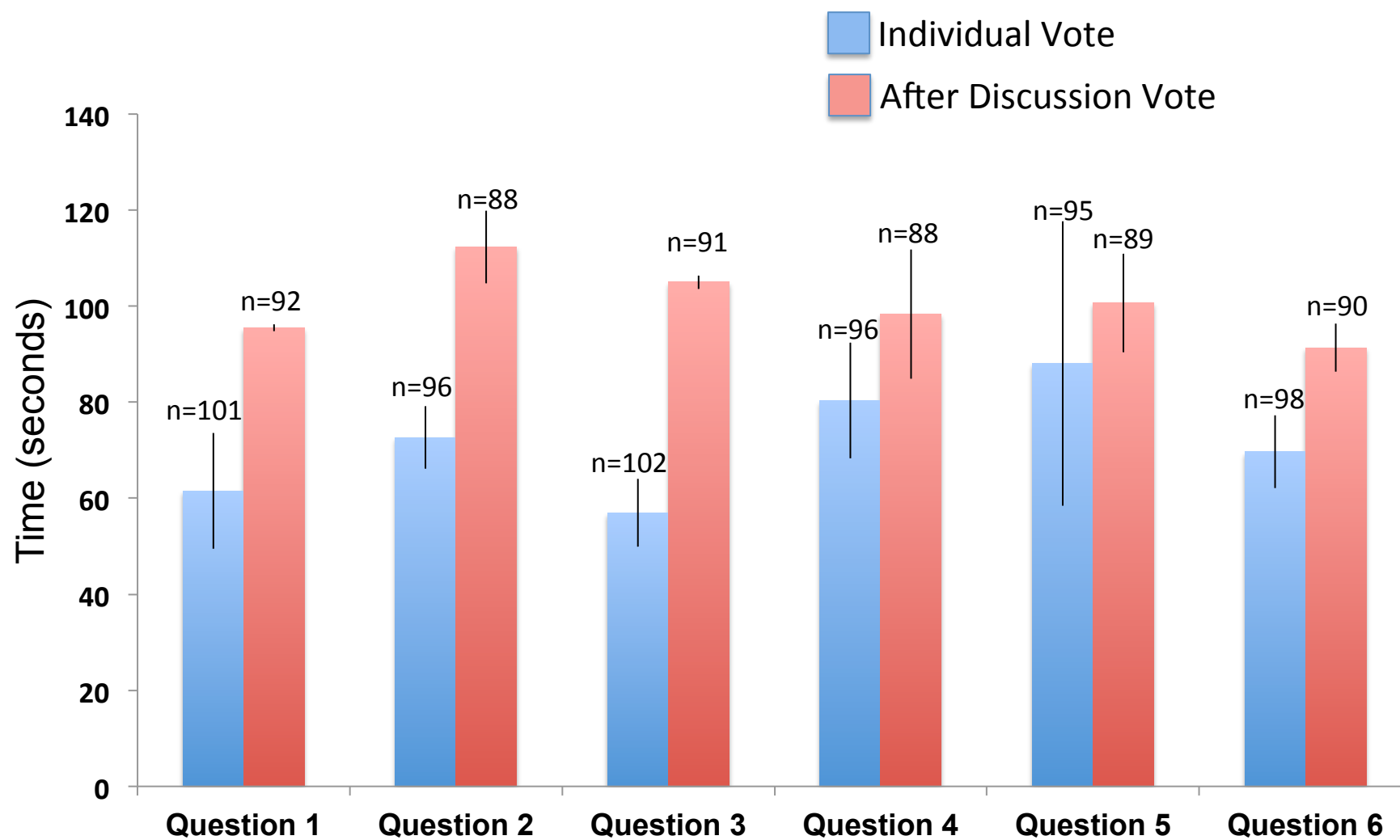

Supplement: Figure S3 — Voting times for individual and after discussion votes for each question. The error bars show STD. The number of blueberry growers participating in each question is also shown. Author S.A. asked the first three questions and author F.D. asked the last three questions. (PDF) [file pone.0047564.s003.pdf]
