# Supplementary material for: Using Peer Discussion Facilitated by Clicker Questions in an Informal Education Setting: Enhancing Farmer Learning of Science
Source: PLoS One. 2012 Oct 15;7(10):e47564. doi: 10.1371/journal.pone.0047564 (PMC3471889; doi:10.1371/journal.pone.0047564)
Supplement: Table S1 — Results of Fisher’s Exact Tests of association between demographic variables and overall trend of blueberry growers in the increase, no change, decrease, and ceiling groups. (DOCX) [file pone.0047564.s004.docx]

Supplemental Table S1.

| Demographic Variable | p-value |
| --- | --- |
| Sex | 0.142 |
| Age | 1.000 |
| Level of education | 0.237 |
| Household income derived from blueberries | 0.181 |
| Role on the farm | 0.863 |
| Time worked with blueberries | 0.220 |
