# Supplementary material for: Using Peer Discussion Facilitated by Clicker Questions in an Informal Education Setting: Enhancing Farmer Learning of Science
Source: PLoS One. 2012 Oct 15;7(10):e47564. doi: 10.1371/journal.pone.0047564 (PMC3471889; doi:10.1371/journal.pone.0047564)
Supplement: Table S2 — Results of the Effect Likelihood Ratio Tests by demographic variable for the Logistic Regression Model. (DOCX) [file pone.0047564.s005.docx]

Supplemental Table S2.

| Demographic Variable | df | χ^2^ | p-value |
| --- | --- | --- | --- |
| Sex | 1 | 0.7318 | 0.3923 |
| Age | 1 | 0.1209 | 0.7280 |
| Level of education | 3 | 0.7654 | 0.8577 |
| Household income derived from blueberries | 2 | 0.3486 | 0.8400 |
| Time worked with blueberries | 2 | 0.5265 | 0.7685 |
